# The Limits of Corporate Social Responsibility: Techniques of Neutralization, Stakeholder Management and Political CSR

**DOI:** 10.1007/s10551-012-1250-5

**Published:** 2012-03-02

**Authors:** Gary Fooks, Anna Gilmore, Jeff Collin, Chris Holden, Kelley Lee

**Affiliations:** 1 Department of Health, University of Bath, Claverton Down, Bath, BA2 7AY UK; 2 Global Health Policy, Centre for International Public Health Policy, School of Health in Social Science, University of Edinburgh, Medical Buildings, Teviot Place, Edinburgh, EH8 9AG UK; 3 Social Policy and Social Work, University of York, Heslington, York, YO10 5DD UK; 4 Faculty of Health Sciences, Simon Fraser University, Room 11322, Blusson Hall, 8888 University Drive, Burnaby, BC V5A 1S6 Canada

**Keywords:** Corporate social responsibility, Political CSR, Techniques of neutralization, Stakeholder management, Corporate political activity

## Abstract

Since scholarly interest in corporate social responsibility (CSR) has primarily focused on the synergies between social and economic performance, our understanding of how (and the conditions under which) companies use CSR to produce policy outcomes that work against public welfare has remained comparatively underdeveloped. In particular, little is known about how corporate decision-makers privately reconcile the conflicts between public and private interests, even though this is likely to be relevant to understanding the limitations of CSR as a means of aligning business activity with the broader public interest*.* This study addresses this issue using internal tobacco industry documents to explore British-American Tobacco’s (BAT) thinking on CSR and its effects on the company’s CSR Programme. The article presents a three-stage model of CSR development, based on Sykes and Matza’s theory of techniques of neutralization, which links together: how BAT managers made sense of the company’s declining political authority in the mid-1990s; how they subsequently justified the use of CSR as a tool of stakeholder management aimed at diffusing the political impact of public health advocates by breaking up political constituencies working towards evidence-based tobacco regulation; and how CSR works ideologically to shape stakeholders’ perceptions of the relative merits of competing approaches to tobacco control. Our analysis has three implications for research and practice. First, it underlines the importance of approaching corporate managers’ public comments on CSR critically and situating them in their economic, political and historical contexts. Second, it illustrates the importance of focusing on the political aims and effects of CSR. Third, by showing how CSR practices are used to stymie evidence-based government regulation, the article underlines the importance of highlighting and developing matrices to assess the negative social impacts of CSR.

## Introduction

Notwithstanding attempts by public institutions such as the European Commission (see, for example, Commission of the European Communities, 2001, 2006) to define corporate social responsibility (CSR), the absence of a widely agreed framework on CSR, which specifies minimum outcome-based standards of social performance, creates an enabling milieu for socially harmful companies which externalise many of their costs to pass themselves off as socially responsible. As well as being a member of Business in the Community (Business in the Community, undated), for example, BAE Systems has published annual CSR reports since 2002 (BAE Systems 2002). This is despite exporting military hardware to oppressive regimes and allegedly using bribes in the sale of arms to developing countries (Leigh and Evans 2009; *R v BAE Systems PLC*, and campaign against the arms trade, undated). Likewise, the world’s four largest private tobacco companies^1^ have highly developed CSR programmes (CSRPs) even though tobacco use is the world’s leading cause of preventable death and global tobacco-related mortality is projected to rise to 8.3 million by 2030 (from 5.4 million in 2005), surpassing the HIV epidemic as the leading cause of premature death (Mathers and Loncar 2006; Gilmore et al*.* 2006, 2009, 2011).

More to the point, research on the use of CSR by the world’s two largest private tobacco companies—Philip Morris (now Philip Morris and Philip Morris International) and British-American Tobacco (BAT)—indicates that their interest in the practice resides largely in its potential to promote policy outcomes that work against public welfare. Specifically, this study suggests that BAT and Philip Morris use CSR politically to prevent the introduction of legally enforceable tobacco control measures which have a proven record of effectiveness in reducing tobacco consumption. In practice, this has involved them using CSR to broker access to public officials, influence the policy alternatives under consideration by elected representatives, break up opposing political constituencies, rebuild tobacco companies’ reputations as providers of reliable information and as a platform for strategic regulation—voluntary forms of corporate governance that are designed to pre-empt formal government regulation which has a proven record of effectiveness in reducing tobacco consumption (Maxwell et al*.* 2000; Collin and Gilmore 2002; World Health Organization 2004; Action Against Smoking and Health, Friends of the Earth and Action Aid 2005; Thomson 2005; Palazzo and Richter 2005; Tesler and Malone 2008; Yang and Malone 2008; McDaniel and Malone 2009; Fooks et al. 2009; Fooks and Gilmore 2011).

In this last respect, existing research shows how BAT, the subject of this study, and other transnational tobacco companies have promoted three key self-regulatory initiatives through their CSR programmes (CSRPs), namely: an international code on marketing which aimed to pre-empt the introduction of legally binding restrictions (Mamudu et al. 2008); ineffective alternatives to public smoking bans based on ventilation and air filtration (Sarnet et al*.*, 2005; Leavell et al*.*, 2006; Muggli et al*.* 2008); and youth smoking prevention schemes—largely ineffective measures aimed at dissuading policymakers of the need for general marketing restrictions (Landman et al. 2002; Assunta and Chapman 2004; Henriksen et al. 2006; Sebrié and Glantz 2007; Apollonio and Malone 2010). By aiming to replace forms of corporate governance that are strongly associated with improved public health outcomes with alternatives for which there is no evidence of effectiveness, these initiatives raise two questions which have potentially far-reaching significance for the contemporary governance of CSR. What do tobacco industry executives think about using CSR against the public welfare and how do these ways of thinking shape, and work within, CSRPs?

## Methodology

This article aims to address these questions using tobacco industry documents made publicly available (http://legacy.library.ucsf.edu/index.html) following litigation in the US to explore how BAT managers’ interpretation of the company’s declining political authority^2^ shaped its CSRP. An iterative approach was taken to searching the archive. Initial searches used broad terms such as social reporting and CSR, and the documents returned from these searches were used to identify narrower search terms, such as the names of key individuals. Searches were performed between April 2008 and July 2009. In total, 143 search terms were used to retrieve 7,987 documents (many of which were duplicates). From these, over 1,784 documents were studied in detail and indexed. Analysis was informed by Forster’s (1994) approach to company document analysis which was complemented using archival techniques discussed by Hill (1993).

There are a number of potential problems in using company documents to construct in-depth accounts of companies’ activities and strategies. Comments contained in emails, presentation notes and strategy papers, for instance, may be capable of multiple interpretations, may not represent the views of all senior managers and can be coloured by personal ambition and office politics. Despite this, they are likely to provide a more reliable guide to corporate decision-makers’ thinking and motivations than interviews which can produce self-serving responses where corporate or professional reputations are at stake. We address some of the problems associated with documentary data by cross-referencing material contained in internal correspondence (including presentation notes) and strategy papers with an analysis of interviews with senior BAT head office staff undertaken by the market research company, Research International (RI). Contracted 2 years prior to the development of BAT’s CSRP, RI was asked to collate ideas about how the company’s public affairs department, CORA (Corporate and Regulatory Affairs) might develop its communications strategy to minimise growing regulatory and litigation risks. RI carried out 12 in depth interviews in December 1996 under the Market Research Society code which is designed to ensure confidentiality and, therefore, optimises the likelihood of responses reflecting honestly held beliefs (Research International, 1997a, pp. 1–5, 1997b).

## Conceptual Approach and Structure of Paper

**Fig. 1:**
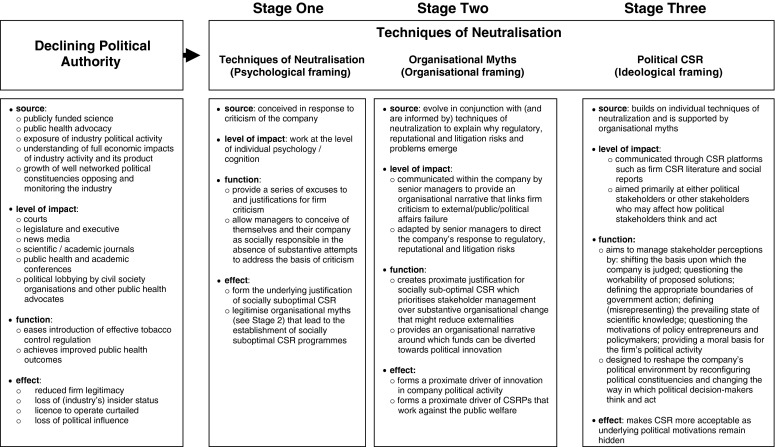
A process model of political CSR

Our analysis draws on and develops Sykes and Matza’s concept of techniques of neutralization, cognitive devices that social actors use to justify, excuse, or in some other way rationalise behaviour that flouts social norms (Sykes and Matza 1957). Sykes and Matza identified five techniques—denial of responsibility, denial of injury, denial of the victim, condemnation of the condemners, and appeal to higher loyalties (see Table 1)—to explain juvenile delinquency; their basic premise being that non-observance of social rules is causally linked to a person’s ability to rationalise transgression by drawing upon a cultural reservoir of motivational vocabularies found in legal principles and dominant cultural norms (Cohen 1993). Subsequent authors have added to Sykes and Matza’s original list of techniques (Table 1) and applied the concept to other offences (see, for example, Cressey 1953; Conklin 1977; Klockars 1974; Minor 1981; Konovsky and Jaster 1989; Szwajkowski 1992; Cohen 1993, 2001; Piquero et al*.* 2005; and Gray 2006) as well as non-criminal behaviour (see, for example, Thompson 1980; McGraw 1990 and Bovens et al. 1999). In this article, we show how corporate decision-makers, when facing a combination of social censure and increased regulatory risk (manifestations of declining political authority), at first reject and then contest the arguments of policy entrepreneurs and reform minded policymakers using techniques of neutralization in a process that we model into three stages (see Fig. 1).

**Table 1:** Techniques of neutralization as they apply to corporate actors

Source	Primary technique	Explanation
Sykes and Matza (1957)	Denial of responsibility	Social actor indicates that harmful behaviour is the result of circumstances or other factors beyond their control
	Condemnation of condemners	Social actor shifts the focus of attention from their own harmful behaviour by raising questions about the motives and behaviour of those who disapprove of their actions
	Denial of harm or injury	Social actor either claims their behaviour is not harmful or disputes the amount of harm caused.
	Denial of the victim	Social actor either claims those harmed deserved it or exploits the fact that victims are physically absent, unknown or a vague abstraction
	Appeal to higher loyalties/authority	Social actor claims behaviour was necessary to conform to the norms of other groups or codes which take precedence over the rules of society or the interests of harmed individuals
Klockars (1974)	Metaphor of Ledger	Social actor characterises their actions as an aberration which is offset by past, ongoing and future good behaviour
Thompson (1980)	Dispersal of blame/transfer of responsibility	Social actors dilute the degree to which they are responsible for harmful behaviour by claiming responsibility for the problem is shared amongst a number of social actors
Minor (1981)	Defence of Necessity	Social actor mitigates blame by claiming their actions were necessary
Bandura (1990)	Dehumanisation of victim	A variant of denying victimhood, where those harmed by social actor’s behaviour are considered not truly worthy of sympathy or compassion
Present paper	Misrepresentation (denial) of the evidence	A variation of denial of harm where corporate actors question the evidence for regulatory intervention by characterising firm arguments relating to regulation as reflecting a fair, balanced or reasonable assessment of the available evidence
	The defence of legality	By pointing to the legality of their product/actions, corporate actors excuse their negative impact on public welfare and justify the existing liberty of action of the company
	For the good of the cause/for the greater good	A variant of appealing to higher loyalties. Corporate actor claims their behaviour was/is for the greater good, producing long-term consequences that serve as a justification of their actions
	Expression of right	A variant of appealing to higher loyalties where corporate actors justify behaviour with reference to (unspecified) universal rights that protect business freedoms
	Protection of the weak	A variant of appealing to higher loyalties where corporate actors claim that behaviour (producing socially suboptimal outcomes) is justified to protect the interests of other, less powerful groups
	Assertion of rationality	A variant of condemnation of the condemners where, by making claims about what is reasonable, fair, constructive and proportionate, the corporate actor questions the reasonableness, fairness, etc., of its detractors
	The world has moved on	Corporate actor claims that shifts in public attitudes rather than own their own behaviour explains public condemnation

In the first stage, reliance by corporate decision-makers on techniques of neutralization provides the basis for declining political authority to be seen as a problem arising from poorly designed and co-ordinated political strategies (rather than a growing awareness of the company’s conduct and its social impacts). During the second stage, corporate decision-makers start attributing the company’s declining political authority to external social actors and proceed to design alternative—and potentially more effective—forms of political management (namely CSR) to annul their impact. Techniques of neutralization expedite this process by providing corporate officials with a resource of justifications that legitimise using CSR politically as a platform of stakeholder management. In the third stage, corporate decision-makers embed techniques of neutralization within the firm’s CSRP to legitimise CSR practices that work against public welfare (by reducing political support for regulatory change). Neutralization is primarily used to produce this last effect by framing opposition to regulatory change as consistent with the broader public interest, creating a distinction between sensible and unreasonable regulation, and building a constructive case for the company’s existing commercial freedoms.

By extending Sykes and Matza’s original concept to illustrate the important role neutralization plays in defending core organisational goals, we not only highlight the political and ideological dimensions of CSR, but also illustrate two interesting characteristics of techniques of neutralization that have not been discussed in the published literature. First, by demonstrating the way in which techniques are used at the corporate level (within BAT’s CSRP), our analysis points to the importance of conceptualising them as political tools and not simply cognitive devices (see Table 1). Second, our discussion also highlights the importance of power in facilitating actors’ ability to neutralise behaviour on a societal level. As we show below, corporate decision-makers seem to be both uncommonly resourceful in developing new techniques from contra-regulatory arguments developed by the tobacco industry over the last 40 years (see Table 1) and, importantly, highly creative in developing new political forms, such as CSR, to legitimise and publicise them. This, we propose, is suggestive of a link between social power and both the range of techniques available to specific classes of social actors and their capacity to broadcast them widely. For juvenile delinquents (and other relatively powerless social actors), neutralization is local, largely internalised and concerned primarily with aligning individual action with broader social norms. In contrast, for corporate actors, neutralization can also be organisational, highly public and concerned with aligning broader social norms with corporate action (see also Yang and Malone 2008).

## Stage One: Declining Political Authority Conceived by Corporate Decision-Makers as a Technical Problem of Political Management

### Declining Political Authority

Historically, the considerable influence tobacco companies enjoyed over public policy making (Nathanson 2005) was predicated on the widespread assumption that the industry made a net contribution to national economies (Holden and Lee 2009). In some countries, this perception was reinforced by political contributions to elected representatives, and considerable investment in traditional forms of corporate political activity aimed, amongst other things, at building strategic alliances with other social actors (Dearlove et al. 2002; Ritch and Begay 2001), covertly funding and generally influencing scientific research (see, for example, Grüning, et al. 2006; Hardell et al. 2007), and shaping the underlying political ideas upon which the tobacco control agenda was set (Cohen et al. 2000). The result, in many countries, was a largely self-regulatory approach to tobacco control (Nathanson 2005). By the 1990s, however, four developments had combined to significantly weaken the industry’s political power.

The first was the widespread acceptance, amongst the public and political élites, that smoking was addictive, was the cause of serious morbidity and mortality and, in the case of second-hand smoke, represented a significant health hazard to non-smokers (see, for example, Marshall 2010). This greatly strengthened the lobbying position of public health advocates (Nathanson 2005). Second, stimulated by research by the World Bank (Warner and Fulton 1995; Warner et al. 1996; World Bank 1999), there was a growing realisation of the negative economic impact of the tobacco industry. Political élites increasingly came to understand that, unlike other industrial sectors, the commercial interests of the tobacco industry were not necessarily consistent with the national economic interest (Holden and Lee 2009). The third concerns the effects of the release of internal tobacco industry documents into the public domain on public perceptions of the industry. Amongst other things, these documents indicated that the industry had understood (but publicly denied) the carcinogenic nature of its product since at least the 1950s and its addictive nature since the 1960s was involved in aggressively defending the freedom to market a product that had been chemically altered to increase its addictiveness; had targeted the young in marketing campaigns; had knowingly promoted ‘low-tar’ cigarettes to offer false reassurance without health benefits and had sought to undermine the scientific debate on the health effects of second-hand smoke (Janofsky 1994; Glantz et al*.* 1996; Test Research 2000; Cummings et al. 2002; Bates and Rowell 2004). These revelations led to cigarette manufacturers being openly demonised as merchants of death who preyed on children and led to reduced trust in the industry amongst policymakers and publics which also greatly strengthened the political influence of public health advocates (U.S. Newswire 1997; Test Research 2000; Carter and Chapman 2003; Nathanson 2005; Palazzo and Richter 2005; Hurt et al. 2009; Haltom et al*.* 2009). Finally, analysis of company documents has increased knowledge of the industry’s heavy reliance on the third party technique in building political constituencies and lobbying policy élites. This has weakened the effect of this strategy by alerting both policymakers and public health advocates to the interests behind political activity relating to the regulation and control of tobacco (Hurt et al*.* 2009; Smith et al*.* 2010; Arnott 2011).

These developments set the conditions for a gradual increase in statutory backed tobacco regulation in the 1990s (see, for example, Aspect Consortium 2004, p. 104) and the decision by the World Health Assembly in 1999 to push ahead with work on the World Health Organisation (WHO) Framework Convention on Tobacco Control (FCTC)^3^ which is currently driving both implementation of tobacco control measures globally and the increasing exclusion of the industry from policymaking (Smith et al. 2009; Fooks et al*.* 2011). Whilst BAT executives recognised these effects as symptomatic of its declining political influence (see, for example, Broughton 1998; BAT 2000a; Marshall 2000a; BAT 2001), they primarily understood the cause of the problem in terms of weaknesses in the company’s political strategy. RI reported that its interviewees held ‘very strong views’ that both BAT and the industry were ‘at fault’ for allowing ‘the current negative climate to be established’, having permitted negative information ‘to frame the debate’ because of a lack of ‘proactivity’ (Research International 1997a, pp. 29, 46 and 49).

### Neutralising Social Censure

Research International’s report strongly suggests that the apparent inability of BAT’s managers to seriously consider and give credence to the work of public health advocates, economists and policymakers which underlay the industry’s decline in political authority was attributable to their heavy reliance on techniques of neutralization. Amongst other things, it recorded ‘a high level of resentment and frustration that individuals faced a wholly negatively framed public debate’, in which staff believed ‘views [had] become seen as facts, when this was not the case’ (Research International 1997a; see also Marshall 2000a). By questioning the evidence upon which arguments for greater tobacco control were based, BAT managers indirectly denied responsibility for tobacco-related harm (denial of responsibility). Further, by highlighting the contrasting regulatory fortunes of other socially harmful industrial sectors with a history of externalising their costs they also implicitly questioned the fairness of increased regulation (condemning the condemners). For example, RI reported that respondents felt it was ‘inconceivable that governments would be as prescriptive with other types of company going about their “normal business practices”’ and claimed that ‘the treatment of the cigarette industry relative to others such as the drinks and car industries was a major point of contention’ (Research International 1997a, p. 32). This point was made particularly strongly in relation to advertising restrictions which managers found ‘infuriat[ing]’ given the ‘inherent unfairness in the way cigarettes, as a legal product, were treated versus other products’ (Research International, 1997a, p.32).

Questioning the fairness of increasing tobacco regulation by emphasising tobacco’s status as a legal product represented the key technique relied upon by managers (the defence of legality). RI noted that, ‘Whilst there was an acceptance of various tobacco/smoking issues being problematic, or delicate, the factor which was mentioned again and again was that BAT [was] marketing a legal product and therefore [had] the right to be active’ (Research International 1997a, p. 30). According to RI, there was ‘a real sense of injustice that tobacco, amongst other legal products, should come in for the criticism it did’. As one of RI’s respondents put it, ‘the simple fact’ was that ‘smoking [was] an activity much as dressmaking which [had] not been declared illegal anywhere in the world’ (Research International 1997a, p. 30). The defence of legality illustrates how corporate officials combine elements of existing techniques to shield themselves from external criticism. First, it implies a diminished awareness of the victim and the harm associated with tobacco (denial of victim and harm). Second, by essentialising the legal status of tobacco, the technique implies the existence of inalienable legal rights to cause harm which take precedence over the harm caused by tobacco (expression of right). Finally, by embedding the defence of legality within a broader narrative of unfairness and inconsistency, it questions the motives of those calling for greater regulation (condemning the condemners).

By providing corporate decision-makers with a cognitive resource to discard the scientific studies, economic analysis and public health advocacy that underlay the firm’s declining political authority neutralization facilitated the development of CSR as a political tool in two important respects. First, it validated poor political management as the primary, or obvious, explanation for declining political authority by effectively invalidating competing explanations. Second, and more importantly, it helped reinforce an organisational culture in which using CSR politically was regarded as ethically sound and legitimate.

## Stage Two: Corporate Decision-Makers Design Alternative Forms of Political Management and Identify External Sources as the Cause of the Company’s Declining Political Authority

Criticism of BAT’s political strategy by RI’s interviewees reflected a view expressed in earlier documents that the firm’s corporate affairs function was disjointed, poorly integrated across BAT’s operating companies and poorly resourced (see, for example, Proctor 1995a). This led to a new department, CORA (Corporate and Regulatory Affairs Department) being established, with responsibility for co-ordinating the company’s political activity.

CORA’s first action plan had simply constituted a synthesis of pre-existing political tactics (BAT 1995; Proctor 1995b; Opukah 1997). Additional assurances by senior management that the new department would be ‘more proactive’ in ‘better positioning the scientific argument’ and providing operating companies with additional support to ‘voice the case’ were given (Herter 1995). However, in the absence of new ideas about how this might be put into effect, it did not constitute a convincing strategy for improving BAT’s political authority. Other research by RI had found that the industry lacked credibility as a source of information on the relationship between smoking and disease (Research International 1997b, p. 83; see also Test Research 2000) and that conventional modes of political activity, which focused on raising doubts about the harm caused by both smoking and second-hand smoke, simply hardened government officials’ perceptions of the industry as untrustworthy (Research International 1997b).^4^ In this context, understanding the company’s loss of political authority as an issue of political management created the organisational impetus to experiment with alternative modes of political activity. RI’s interviewees specifically argued for measures that provided ‘an alternate stance…rather than an oppositional one’ (Research International 1997a, p. 37) and suggested that this could be achieved by repackaging the firm’s ‘philanthropic activities’ and experimenting with tactics that centred on improving the company’s reputation (Research International 1997a, p. 38).

RI made this a key recommendation of its report and it was later embedded in CORA’s strategic objective which aimed to generate a perception of BAT as a responsible company in a controversial industry (Prideaux and Smith 1999; BAT 2000b). This approach was judged to be capable of producing two key benefits. First, it was considered to be a potentially powerful agenda setting device (Research International 1997a; see also Brendan Brady, former director of corporate and regulatory affairs for BAT Australasia cited in Burton and Rowell 2002 and Prideaux 2000). This was particularly thought to apply in developing economies where, according to one RI interviewee, greater levels of ignorance over tobacco and health ‘issues’ gave BAT a ‘much better chance of being able to manage the situation (Research International 1997a, p. 30)’. Second, it was regarded as an effective way of protecting employees from criticism by friends and family of deceased smokers they met outside of work (Research International 1997a, pp. 33–34, 36). Some staff were unapologetic, refusing to express contrition on the basis of the considerable time and effort they put into their work (Research International 1997a, p. 34). However, most of RI’s interviewees wanted the company to take a more active role in managing the impressions of others (Research International 1997a, p. 35; Goffman 1959). BAT staff, it seems, saw CSR as a resource they could draw on to defend themselves in social settings by maintaining their preferred definition of the company and, therefore, themselves.

To understand how these aims were put into effect, some understanding of the link between techniques of neutralization and organisational myths is required. We use the term organisational myth in this discussion to refer to a narrative form or frame which defines problems, identifies their causes, makes moral judgements about them and offers solutions for their resolution (Entman 1993). They play a central role in the creation of organisational cultures and are either actively developed by senior managers, in the context of managed communication to shape the shared beliefs and values of employees (Kaye 1995) or simply emerge through shared stories and accounts independently of managed communication (Cohen and Prusak 2001). BAT documents suggest that CORA employees repackaged several techniques of neutralization into a myth of political persecution which was then used in the company’s strategy documents and managed communication to both legitimise and guide the development of, the company’s CSRP.

The essence of the myth was that declining political authority was attributable to the potency of public health advocates. Advocates were characterised as voluble, extreme and highly effective at securing public consent for new forms of tobacco control using ‘emotionally charged’ arguments (Marshall 2000a, p. 2; BAT 2000c, p. 4; Weishaar et al*.* under review). The widespread scepticism BAT encountered as a result was described as an ‘emotional wall’—a metaphor used by CORA officials to encapsulate BAT’s marginalisation in policymaking and public debate (Marshall 2000a, p. 5).

By defining the public as passive recipients of information, and public health advocates as emotive and extreme, BAT’s management collapsed two mutually reinforcing ideas into the myth—that the mainstream case against tobacco was non-objective and unscientific and that the company’s position was reasonable and sensible (Marshall 2000b). These ideas (which implicitly denied the harm associated with tobacco products, condemned the company’s condemners and asserted the company’s own rationality—see Table 1) were applied to all stakeholders seeking to use legal and political channels to hold BAT to account. Thus, judges and plaintiff lawyers were accused of attempting to ‘rig trials’, the WHO was portrayed as promoting extreme and unreasonable regulation (Weishaar et al*.* under review) and the House of Commons Select Committee investigating the tobacco industry was dismissed as ‘an ill informed and poorly briefed “kangaroo court” convened to harass the industry in an attempt to harden public opinion and enable them to push through tougher regulation’ (Marshall 2000a). By maintaining these myths and simultaneously asserting that the company’s ‘primary responsibility’ of commercial success was good for the countries in which it operated (BAT 1998) (the good of the cause), BAT managers essentially manufactured the moral justification for CSR to be used politically to align the views of external stakeholders with the commercial interests of the firm.

In practice, the company sought to achieve this using stakeholder engagement to reduce the influence of public health advocates over stakeholders’ opinions about tobacco regulation (BAT 2000c). Engagement was organised around six ‘reputation management initiatives’, which covered issues such as consumer information, ‘responsible marketing’ and youth smoking prevention. Stakeholder analysis, planning, engagement and communication were identified as central to developing these initiatives (BAT, undated-a). An extensive stakeholder mapping exercise was planned (BAT 2000d). This involved identifying individuals and institutions who were likely to have ‘the greatest impact on BAT’s ability to do business’ and categorising them according to whether they were decision-makers (actors having an immediate impact on ‘BAT’s ability to achieve long-term growth’) or influencers (actors able to exert influence over the views and activities of decision-makers or influential groups such as media and pressure groups). Once identified, stakeholders were surveyed to assess their relative enmity towards BAT and the potential flexibility of their views (BAT 2000d, e). The results were converted to a numerical rating, reflecting stakeholders’ ‘attitude’ and ‘flexibility’, and then integrated into a series of maps to identify ‘decision-makers’ and ‘influencers’ who should be ‘strategically *managed* from the centre’ [*emphasis added*] (BAT 2000d, g, h, undated-b, undated-a, undated-c).

‘Managed’ can take on different meanings, depending on the context in which it is applied. BAT documents suggest that it implied a highly active process of impression management. A key element of this was to create divisions within alliances favouring evidence-based tobacco control and reduce support for them amongst the general public (BAT 2000c, d, f, g, h, undated-b, undated-a). In the section below, we focus on how the company uses neutralization in its Framework for CSR (hereafter referred to as Framework) as an ideological or framing tool^5^ to produce this effect.

## Stage Three: Techniques of Neutralization, Issue-Framing and Political CSR

Developed in the context of formal dialogue with stakeholders (see BAT 2002, 2003, 2004, 2005), the Framework—a lengthy document running to just under 5,000 words which constitutes BAT’s roadmap for corporate responsibility—is split into three levels. The first level comprises three organising ‘business principles’ [‘mutual benefit’^6^ (Table 2) ‘responsible product stewardship’^7^ (Table 3) and ‘good corporate conduct’^8^], which correspond to a series of ‘core beliefs’ (the second level) that provide the basic structure of the Framework (Tables 2, 3). The third level contains a series of proposed actions and suggested responsibilities for BAT, policymakers and NGOs that are presented as a blueprint for socially responsible tobacco production, distribution and consumption (Tables 2, 3).

**Table 2:** Techniques of neutralization contained in proposed actions and suggested responsibilities associated with the principle of mutual benefit (BAT, undated-d)

Level one (core beliefs)	Level two (proposed actions and suggested responsibilities)	Technique of neutralization
We believe in creating long-term shareholder value	‘[BAT] is owned by shareholders whose rightful expectation is that we should grow its profitability by…competing effectively for market share…’	Expression of right Appeal to higher loyalties
	‘we see it as the responsibility of governments…to uphold consumers’ rights and freedoms of choice’	Condemnation of condemners
	‘…we will…work with governments to preserve the rights of…adults…to choose the products and brands they prefer’	Protection of the weak Appeal to higher loyalties
	‘we will…work with all relevant stakeholders for preservation of opportunities for…adults to consume tobacco products’	Protection of the weak Appeal to higher loyalties
	‘we see it as the responsibility of governments…to uphold our right to conduct a legal and competitive business’	Defence of legality Expression of right
	‘we see it as the responsibility of governments…to make balanced decisions based on sound evidence’	Condemnation of condemners
	‘we will…work with governments to preserve the rights of informed adult consumers’	Protection of the weak Appeal to higher loyalties
	‘we share a role with other parts of society in respecting the rights and freedoms of informed adults to consume tobacco products’	Protection of the weak Appeal to higher loyalties
	‘tobacco products are legal, significant demand for them exists…and informed adults have rights to consume them and to choose the brands they prefer’	Protection of the weak Appeal to higher loyalties Defence of legality
	‘we have a role in helping to preserve our consumers’ rights’	Protection of the weak Appeal to higher loyalties
We believe in adding value to the communities in which we operate	‘as corporate citizens…our companies have a role in investing in local economic, social and cultural development…host communities can benefit from this…’	For the good of the cause
	‘…it [is] the role of governments and regulatory authorities to create environments where business can thrive &…contribute to local economic, social & environmental development’	Condemnation of condemners
	‘we will assist by explaining to governments and regulatory authorities the conditions within which business can thrive’	For the good of the cause
We believe in engaging constructively with our stakeholders	‘we see it as the responsibility of our stakeholders, including our critics, to engage constructively with us’	Condemnation of condemners Transfer of responsibility

**Table 3:** Techniques of neutralization associated with the principle of responsible product stewardship (BAT, undated-d)

Level two (core beliefs)	Level three (proposed actions and suggested responsibilities)	Technique of neutralization
We believe in the appropriate taxation of tobacco products and the elimination of illicit trade	‘we see it as the responsibility of governments and multilateral organisations to establish workable fiscal regimes and economic policies that do not create the conditions for illicit trade, and to implement and enforce effective legislation and strong border controls directed towards combating illicit trade’	Transfer of responsibility
We believe in regulation that balances the interests of all sections of society, including tobacco consumers and the tobacco industry	‘we…reserve our right to challenge regulation which is disproportionate and undermines the fundamental protections that legal systems afford us’	Condemnation of condemners Expression of right
	‘we believe that regulation should also respect the rights of adult consumers to continue making informed choices about legal products and the industry’s ability to operate and compete’	Protection of the weak Appeal to higher loyalties
	‘we will…seek solutions that balance the interests of all concerned. We should work together with other parts of society to try to ensure that tobacco regulation is balanced and workable’	For the greater good
	‘we see it as the responsibility of governments to ensure that tobacco regulation is evidence based, proportionate and aligned with a transparent and realistic objective’	Assertion of rationality For the greater good
	‘we will support governments by…promoting the view that tobacco regulation should be based on a balanced consideration of the interests of all parts of society’	Protection of the weak Appeal to higher loyalties
	‘we will support governments by.. advocating that tobacco regulation should be enforced consistently’	Condemnation of condemners
	‘we will…work with governments on their issues of concern, advocate respect for the rights of adult consumers’	Exercise of right Appeal to higher loyalties
We believe that underage people should not consume tobacco products	‘we see it as the responsibility of society as a whole, and specifically of governments, educators and parents to reduce underage use of tobacco products’.	Transfer of responsibility Dispersal of blame
We believe that public smoking should be approached in a way that balances the interests of smokers and non-smokers	‘environmental tobacco smoke (ETS) is often a source of annoyance to non-smokers and smokers alike and is considered by some public health authorities to be a health concern’	Misrepresentation of evidence
	‘we think a sense of proportion needs to be maintained’	Assertion of rationality Misrepresentation of evidence
	‘…outright public and workplace smoking bans…prejudice the rights of smokers to consume a legal product and, in our view, are generally unnecessary’	Assertion of rationality Misrepresentation of evidence
	‘there are solutions to the problem of ETS that allow the accommodation of smokers and non-smokers’	Misrepresentation of evidence
	‘our role is to make clear our views on this issue, to suggest solutions that accommodate smokers and non-smokers’	Protection of the weak
	‘our role is to…call for regulations that are soundly based’	Misrepresentation of evidence
	‘we will…promote sensible public and workplace smoking arrangements with a range of stakeholders, including governments and building managers’	Misrepresentation of evidence
	‘we see it as the role of governments and society to take the lead in approaching public smoking in a balanced and proportionate way, based on sound scientific evidence’	Assertion of rationality Misrepresentation of evidence

Until 2006, the Framework was published in BAT’s social reports and selectively quoted in response to points raised by stakeholders (see, for example, BAT 2005). At the time of writing, it is no longer produced in the company’s new Sustainability Reports, but remains available on the company’s website (BAT, undated-d). The core beliefs (second level), proposed actions and suggested responsibilities (third level) relating to the principles of mutual benefit and responsible product stewardship contain a number of techniques of neutralization that BAT has drawn on to validate political activity aimed at opposing the introduction of widely supported (and evidence based—see, for example, Hopkins et al. 2010) forms of tobacco control (see, for example, BAT, undated-e; BAT, undated-f; BAT, undated-g; BAT, undated-h). By prescribing how the relative strengths of competing approaches to controlling tobacco products should be conceived by politically influential stakeholders (such as policy élites and civil society groups), these parts of the Framework play an important role in BAT’s efforts to shape the agenda for tobacco control (Brendan Brady, former co-director of CORA BAT Australasia, cited in Burton and Rowell 2002; see also Millson 1999; McCombs et al*.* 1997).

To illustrate this effect, we have extracted the techniques of neutralization embedded in the principles of ‘mutual benefit’ and ‘responsible product stewardship’ in Tables 2 and 3. By collectively promoting proprietary and other economic interests over-and-above public health, the techniques we identify work to justify BAT’s existing commercial freedoms (hereafter described as regulatory conservatism) and define political activity that works against public welfare as socially responsible.

Regulatory conservatism is defended partly in terms of the importance of protecting shareholders’ and smokers’ rights (expression of right/appeal to higher loyalty/protection of the weak) and partly in terms of the economic contribution that BAT’s investment makes to ‘host communities’ (for the good of the cause). The internal logic of these propositions is maintained by relying on other less explicit techniques that ignore the epistemological complexities involved in building a case for the manufacture and sale of tobacco as socially responsible. Hence, the idea that BAT’s investment benefits communities glosses over the net economic harm of tobacco manufacture (misrepresentation of the evidence/denial of harm) (World Bank 1999) and the moral validity of both claims is underwritten by misleading references to the health effects of second-hand smoke as a ‘source of annoyance’ and of a health concern only to ‘some public health authorities’ (misrepresentation of the evidence/denial of harm). Finally, by asserting the company’s privileged access to rationality (assertion of rationality) through constant use of terms such as balanced and proportionate, the Framework formalises an intellectual basis for the faux distinction between sensible and unreasonable regulation—a contrast BAT has consistently used to support regulation in principle but contest it in practice (BAT (Mauritius) 2004; BAT 2000i).

BAT defends socially suboptimal political activity by characterising it as a crucial tool for achieving a balanced approached to health policy which protects the interests of shareholders and smokers. By requesting balance, the company effectively represents itself as a neutral broker of equally competing interests and, therefore, its political activity as arbitration, rather than partisan lobbying. Furthermore, by emphasising the rights and effective disenfranchisement of shareholders and smokers BAT also links its political work to the defence of minority interests and inalienable rights (protecting the weak/expression of right). The persuasive power of this approach derives partly from the fact that it inverts the nature of the relationship between the company and its customers and partly from the fact that it sets the company’s political activity within the traditions of classic liberalism. Smokers, particularly those who begin smoking before adulthood^9^ and who express a desire to quit,^10^ are victims in the conventional meaning of the term in that their health and well-being are negatively affected by an addiction developed before reaching an age when they are deemed capable of giving informed consent. By reclassifying them as victims of state intervention,^11^ BAT effectively negates their status as victims of tobacco manufacture and marketing, and reinforces its preferred definition of political activity as protective and socially progressive. Moreover, by referring, albeit implicitly, to the importance of protecting fundamental rights from state power (Ashcraft 1987), the approach also binds BAT’s political activity to the broader egalitarian aspirations of liberal politics and by describing it as a ‘support’ to governments, presents it as a free subsidy to policy makers.

Drawing out the considerable range of techniques of neutralization embedded within BAT’s Framework, points to the defensive nature of CSR for companies, like BAT, which face acute regulatory risks. Importantly, it also illustrates the way in which CSR practices provide a platform for highly externalising companies to reassert both the moral primacy of their shareholders’ economic interests, and the legitimacy of political activity aimed at maximising the return on capital invested irrespective of its social impacts. Moreover, by highlighting BAT’s inventiveness in stretching the existing range of techniques covered in the academic literature (see Table 1), it also points to the ability of powerful economic actors to optimise the political value of neutralization.

BAT’s innovation in using techniques of neutralization appears to be built on its ability to develop arguments used in previous political campaigns by the tobacco industry (see, for example, Arno et al. 1996; Menashe and Siegel 1998; Cohen et al*.* 2000; Ong and Glantz 2001; Apollonio and Bero 2007; Apollonio and Bero 2009) and repackage them as positive principles which ‘seek solutions’ and indicate a willingness to ‘work with’ and provide ‘support’ to government (BAT, undated-d).^12^ This highlights BAT’s ability to collectivise the resources of the industry and exploit its historical investment in public relations (see, for example, Ong and Glantz 2001; SourceWatch, undated). Further, its ability to neutralise through positive statements is a direct result of its capacity to invest in CSR practices, such as social reporting, which exist alongside, and independently, of formal policy making networks. One consequence of this is that BAT can assert arguments, based on its Framework for CSR, without engaging directly with the evidence-based arguments of public health professionals and reform minded public officials. The effect is a sort of highly managed monologue, in which BAT selectively summarises opposing arguments (see Diethelm and McKee 2009 for an analysis of some of the techniques involved in this process) and sets out the appropriate role of government and, therefore, appropriate scope of government intervention (see BAT 2004 and 2005). In short, the ability to fund CSR practices provides BAT with a platform to amplify excuses and justifications for socially harmful behaviour in a way that appears constructive and socially progressive.

## Discussion and Conclusion

Research on the strategic dimensions of CSR and corporate philanthropy has historically concentrated on measures that further the immediate interests of the corporation, whilst also producing social benefits (Burlinghame and Young 1996; Saiia 2001; Porter and Kramer 2002; Orlitzky et al. 2003; Saiia et al. 2003; Thorne et al. 2003; Campbell and Slack 2008; Heslin and Ochoa 2008). There are some exceptions to this focus in the public health literature where studies examine CSR practices that work against the broader public welfare (see, for example, Landman et al*.* 2002). However, these studies leave unexplored how corporate decision-makers reconcile the obvious conflicts involved in using CSR in this way and how these modes of thinking then shape CSR practices. Our analysis explores these questions by focusing on how the concept of neutralization helps make sense of BAT’s CSR programme. The study supports two broad conclusions.

First, it shows how neutralization can resolve contradictions in the way in which a firm’s management makes sense of the two quite different concepts of stakeholder engagement and stakeholder management and create the conditions necessary for them to ignore the ethical implications of using CSR as a mode of regulatory management. By illustrating how BAT’s management dismissed social censure of tobacco manufacturers as scientifically ill-informed and driven by extreme organisations and reform minded policymakers, the concept of neutralization helps us to understand why the firm’s CSR practices only superficially address the important issue of tobacco consumption (Action Against Smoking and Health 2005). Despite the nature of the company’s business, and a long history of commercial dishonesty ranging from silence over the addictive nature of nicotine to complicity in tobacco smuggling (see, for example, Campaign for Tobacco Free Kids 2001; Campbell 2004; Bates and Rowell 2004; Beelman et al*.* 2000), heavy reliance on neutralization meant that BAT’s management never doubted that they were working for a socially responsible corporation trading in a socially responsible way. ‘Essentially’, as RI’s report put it, ‘staff believed that British-American Tobacco was a good international citizen currently, but had failed historically to project itself as such’ (Research International 1997a, p. 39). This not only set the background to efforts aimed at focusing CSR practices on issues such as reputational benefit, political access and opportunities for constituency building, rather than on the company’s impact on local communities, customers or the environment (see, for example, Oliver 1998), it also meant that fundamental changes in either the company’s business model or levels of investment in social activity were unnecessary. This is consistent with strategy papers indicating that CORA staff believed BAT was already investing heavily in socially responsible activity, but was not doing enough—through branding and publicity—to convey this to a sceptical public (BAT 2000e).

The second conclusion concerns the potential ideological effects of neutralization and centres on the way in which neutralising techniques relied on during the development of the company’s CSRP spill over into CSR practices and underpin their use as a political tool. Specifically, neutralization was central to how corporate decision-makers used CSR as a framing device to transmit a specific view of the problems relating to tobacco and its control that redefine the range of legitimate solutions (Entman 1993). In practice, this has involved BAT using its CSRP to reject further regulation, provide a moral framework for defining political activity that works against public welfare as socially responsible and create an intellectual basis for the contrived distinction between sensible and unreasonable regulation.

Company documents suggest a key aim of this approach was to break up and, therefore, weaken opposition to BAT’s efforts to optimise its commercial freedom and that it was guided by two main impulses: the need for BAT employees to reconcile the nature of their work with the perceptions of friends, family and acquaintances, as well as their own sense of self; and the commercial pressure to produce ‘potential benefits’, such as ‘increased marketing freedoms, increased self-regulation, greater intention to buy’ (BAT, undated-i; Canning 1999). This second driver illustrates the importance of identifying and quantifying the negative effects of CSR practices when assessing their broader social impact.

Our analysis has potentially important implications for the capacity of voluntary forms of corporate governance, such as social reporting and stakeholder dialogue, to reduce business’ negative social and environmental impacts. The extent to which these CSR practices can bring corporate activity more closely into line with the broader public interest is predicated on their capacity to make corporations more accountable to stakeholders whose interests are not purely confined to optimising returns on capital invested. Our analysis, in contrast, strongly suggests that stakeholder dialogue (and, therefore, social reporting) is primarily a defensive practice aimed at preventing stakeholders from forcing change on companies through formal government intervention. In this sense, our data indicate that the ‘managerial capture’ of CSR (Puxty 1991; Owen et al. 1997; Owen et al. 2000 and O’Dwyer 2003) is an ongoing and active process which takes effect in the day-to-day practices of stakeholder engagement. Whilst strong commercial drivers underlie this approach, it is facilitated by corporate decision-makers’ reliance on neutralization which provides them with a cognitive repertoire for responding to some aspects of stakeholder opinion, but not others—a vital stage in the process of subscribing to stakeholder engagement in principle, but using it in practice as a means of managing regulatory risk.

The extent to which our analysis helps to explain the finding of other studies on the appropriation of CSR by corporate managers (see, for example, Larrinaga-Gonzalez and Bebbington 2001; O’Dwyer 2003; Corporate Watch 2006; Crowther 2006), and addresses the broader limitations of CSR as a means of aligning business activity with public welfare, is ultimately dependant on whether we make sense of large tobacco companies as a special case. Tobacco is the only product that kills when used as intended. Further, the mortality rate of long-term tobacco use is unusually high (Centers for Disease Control and Prevention 2002). Nevertheless, the behaviour of tobacco company executives is not unique. Litigation against corporations tells us that company executives in other sectors such as mining (Rosner and Markowitz 1994), pharmaceuticals (Abraham and Davis 2006), asbestos (Tweedale 2000; McCulloch and Tweedale 2008), chemicals (Pearce and Tombs 1998) and oil (Rosner and Markowitz 2006), have been dishonest about the degree of harm their businesses cause. Furthermore, companies in these sectors have misrepresented scientific knowledge to reduce regulatory and litigation risk (Tucker 2006; McGarity and Wagner 2008; Wagner and Steinzor 2006; Hoggan and Littlemore 2009). In fact, many of the political strategies used by the tobacco industry are common tools of the public relations industry (Stauber and Rampton 1995), and it is therefore not surprising to find neutralization being linked to CSR practices in other industrial sectors (Baumberg, undated). If, in rejecting tobacco industry exceptionalism, we assume that the findings summarised in this article are capable of being extended to other companies, the prognosis for voluntary forms of CSR as a means of managing the increasing costs of business activity to public health and the environment seems limited, at least where socially responsible business practices diminish company earnings. If managers typically start from an assumption that a firm is already socially responsible, and that criticism directed at the firm is unjustified or politically motivated, then they are more likely to come to regard CSR as a public relations tool or device for managing regulatory environments than as a medium for undertaking meaningful change.

## Abbreviations

ASH: Action against smoking and health
BAT: British-American tobacco
CORA: Corporate and regulatory affairs department
CSR: Corporate social responsibility
CSRP: Corporate social responsibility programme
FCTC: Framework convention of tobacco control
NGO: Non-governmental organisation
RI: Research international

## Footnotes

1 Outside of China, these four companies—British American Tobacco (BAT), Philip Morris International, Japan Tobacco International and Imperial Tobacco—constitute an effective global oligopoly with a combined global market share (inclusive of China) of just over 50% (Hedley 2007; Callard 2010). Smoking causes fatal and disabling disease, and, compared with other risky behaviours, the risk of premature death is extremely high. Half of all long-term smokers will eventually be killed by tobacco, and of these, half will die during productive middle age, losing 20–25 years of life. The choice to smoke differs from the choice to buy other consumer goods in three key ways. First, many smokers are not fully aware of the high risks of disease and premature death of smoking. In China in 1996, for example, 61% of smokers questioned thought that tobacco did them ‘little or no harm’. In high-income countries, smokers know they face increased risks, but they judge the size of these risks to be lower and less well established than non-smokers, and they also minimise the personal relevance of these risks. Second, smoking (which is usually started in adolescence or early adulthood) is addictive. Most new recruits underestimate the risk of becoming addicted to nicotine. In high-income countries, individual attempts to quit have low success rates: of those who try without the assistance of cessation programmes, about 98% will have started again within a year. In low- and middle-income countries, quitting is rare. As a result, they seriously underestimate the future costs of smoking. Third, smoking imposes health and financial costs on non smokers. In high-income countries, for example, smoking-related healthcare accounts for between 6 and 15% of all annual healthcare costs. In any given year, smokers’ healthcare costs on average exceed those of non-smokers’. Further, reviews in high-income countries suggest that smokers’ lifetime costs are higher than non-smokers, despite their shorter lives (World Bank 1999; Tobacco Free Initiative 2006; Office for National Statistics 2009 and 2011).

2 We use political authority rather than political power to convey the decreasing acceptance amongst policy élites of the legitimacy of tobacco industry political power.

3
http://www.who.int/features/2003/en/08_gallery2.html, accessed 20.10.09.

4 Some BAT staff also believed it had the effect of stiffening their resolve to introduce statutory regulation (Marshall 2000b, p. 3).

5 A frame is defined here as a means of packaging and positioning an issue in terms of defining the problem, diagnosing its causes making normative judgments and suggesting remedies (Entman 1993). The effects of issue-framing on public opinion are well documented (Vaughan and Seifert 1992; Jacoby 2000; Schneider and Jacoby 2005), but it is also thought to play a central role in the formation of public health policy. In a review of the legislative and regulatory history of tobacco control in the USA, for example, Jacobson et al*.* (1997) found that policymakers’ ability to introduce tobacco control legislation was closely related to how the legislative debate was framed, with efforts to enact legislation stalling when the debate shifted to issues about personal freedoms (see also Wallack et al*.* 1993).

6 This is defined as the basis on which the company builds its relationships with its stakeholders. It states that the company is primarily in the business of building long-term shareholder value and it emphasises the belief that the best way to do this is to seek to understand and take account of the needs of all the company’s stakeholders.

7 This is defined as the basis on which the company meets consumer demand for a legal product that is a cause of serious diseases. It states that the company’s products and brands should be developed, manufactured and marketed in a responsible manner and emphasises that the company aspires to develop tobacco products with mass appeal that will, over time, be recognised by scientific and regulatory authorities as posing substantially reduced risks to health.

8 This emphasises the importance of observing high standards of behaviour and integrity throughout the company’s operation.

9 In the UK, an estimated 65% of ever smokers start smoking before the age of 18 (National Statistics 2000).

10 In the UK, the proportion of smokers who express a desire to quit consistently exceeds two-thirds (Lader 2007; Office for National Statistics 2011).

11 In practice, a majority of current smokers in the UK broadly supported public smoking bans (Lader 2007).

12 The company’s justification of socially harmful action is, as such, almost invariably oblique and implied. Thus, in calling on governments to approach public smoking in a balanced and proportionate way (based on sound scientific evidence), the Framework does not explicitly isolate existing approaches as unbalanced or disproportionate to the underlying risks but when interpreted in conjunction with other propositions (such as the assertion that ETS is only considered to be a health concern by some public health authorities), this is its natural implication.

## References

[CR5] Abraham J, Davis C (2006). Testing times: The emergence of the practolol disaster and its challenge to British drug regulation in the modern period. Social History of Medicine.

[CR6] Action against smoking and health, friends of the earth and action aid. (2005). *BAT in its own words*, http://www.foe.co.uk/resource/reports/bat2005.pdf. Accessed May 11, 2008.

[CR7] Apollonio DE, Bero LA (2007). The creation of industry front groups: The tobacco industry and “get government off our back”. American Journal of Public Health.

[CR8] Apollonio DE, Bero LA (2009). Evidence and argument in policymaking: Development of workplace smoking legislation. BMC Public Health.

[CR9] Apollonio DE, Malone RE (2010). The “We Card” program: Tobacco industry “youth smoking prevention” as industry self-preservation. American Journal of Public Health.

[CR10] Arno PS, Brandt AM, Gostin LO, Morgan J (1996). Tobacco industry strategies to oppose federal regulation. JAMA.

[CR11] Arnott, D. (2011). *Tobacco industry lobbying on display ban: A present day case study from the UK*. European Conference on Tobacco or Health, Amsterdam, 28–30.03.11.

[CR12] Ashcraft R (1987). Locke’s two treatises of government.

[CR13] Aspect Consortium (2004) Tobacco or Health in the European Union; Luxembourg: European Commission, Office for Official Publications of the European Communities. Accessed April 12, 2011 from http://ec.europa.eu/health/ph_determinants/life_style/Tobacco/Documents/tobacco_fr_en.pdf.

[CR14] Assunta M, Chapman S (2004). Industry sponsored youth smoking prevention programme in Malaysia: A case study in duplicity. Tobacco Control.

[CR2] BAE Systems. (2002). *Corporate social responsibility review 2001*. Accessed Feb 26, 2010 from http://www.baesystems.com/BAEProd/groups/public/documents/bae_publication/bae_pdf_ccomms_cr01.pdf.

[CR16] Bandura A, Kurtines WM, Gewirtz JL (1990). Social cognitive theory of moral thought and action. Handbook of moral behaviour and development: Theory, research and applications.

[CR37] Bates, C., & Rowell, A. (2004). *Tobacco explained…The truth about the tobacco industry…in its own words*, WHO Tobacco Control Papers, Center for Tobacco Control Research and Education, UC San Francisco, Accessed Aug 10, 2010 from http://www.escholarship.org/uc/item/9fp6566b.

[CR17] BAT. (1995). *BATCo corporate affairs company planning guidelines*. Bates numbers: 800269455–800269456. Accessed may 13, 2008 from http://legacy.library.ucsf.edu/tid/zav14a99.

[CR18] BAT. (1998). *Survey on corporate social responsibility at British-American tobacco company limited*. Bates numbers: 322121425–322121436, 322121425. Accessed Feb 2, 2011 from http://legacy.library.ucsf.edu/tid/xoz82a99.

[CR19] BAT. (2000a). *BAT cora partnership for change communications direction 29th september 2000.* Bates numbers: 325046507–325046510. Accessed Sep 13, 2008 from http://legacy.library.ucsf.edu/tid/euz82a99.

[CR20] BAT. (2000b). *The CORA roadmap: CORA strategic steering group*. Bates numbers: 325136086–325136276. Accessed May 12, 2008 from http://legacy.library.ucsf.edu/tid/dew70a99.

[CR21] BAT. (2000c). *The CORA roadmap: CORA regional meetings-june 2000.* Bates numbers: 325419015–325419028. http://legacy.library.ucsf.edu/tid/xzz24a99.

[CR22] BAT. (2000d). *BAT: Stakeholder analysis*. Bates number: 325049596–325049599. Accessed May 10, 2008 from http://legacy.library.ucsf.edu/tid/dzo14a99.

[CR23] BAT. (2000e). *Corporate and regulatory affairs: Company plan 1999–2001*. Bates numbers: 321324291–321324477. 25.09.98. Accessed Dec 12, 2009 from http://legacy.library.ucsf.edu/tid/eip73a99.

[CR24] BAT. (2000f). *Cross regional CORA conference on 28–30 June 2000*. Bates numbers: 760072400–760072631. Accessed Oct 20, 2009 from http://legacy.library.ucsf.edu/tid/alj55a99.

[CR25] BAT. (2000g). *Stakeholder mapping and classification*. Bates numbers: 760024496–760024549. Accessed May 11, 2008 from http://legacy.library.ucsf.edu/tid/anj45a99.

[CR26] BAT. (2000h). *Stakeholder mapping and classification*, Bates numbers: 760024550–760024597. Accessed may 12, 2008 from http://legacy.library.ucsf.edu/tid/bnj45a99.

[CR27] BAT. (2000i).* British American tobacco proposes ‘quantum leap’ for sensible tobacco regulation*. Accessed Dec 15, 2009 from http://www.bat.com/group/sites/uk__3mnfen.nsf/vwPagesWebLive/DO533JSX/$FILE/medMD533L8U.pdf?openelement.

[CR28] BAT. (2001). *The case for engagement*. Bates numbers: 325047041–325047059. Accessed May 12, 2008 from http://legacy.library.ucsf.edu/tid/ino14a99.

[CR29] BAT. (2002). Social report 2001/2002, Accessed Mar 15, 2009 from http://www.bat.com/group/sites/uk__3mnfen.nsf/vwPagesWebLive/DO6RZGHL/$FILE/medMD5PRUNF.pdf.

[CR30] BAT. (2003). Social Report 2002/2003, Accessed Mar 15, 2009 from http://www.bat.com/group/sites/uk__3mnfen.nsf/vwPagesWebLive/DO6RZGHL/$FILE/medMD5PVKV6.pdf?openelement.

[CR31] BAT. (2004). Social Report 2003/2004, Accessed Mar 15, 2009 from http://www.bat.com/group/sites/uk__3mnfen.nsf/vwPagesWebLive/DO6RZGHL/$FILE/medMD626PBK.pdf?openelement.

[CR3] BAT (Mauritius). (2004). Social Report, Accessed Dec 15, 2009 from http://www.bat.com/group/sites/uk__3mnfen.nsf/vwPagesWebLive/54102427D9A9BADAC12573140052EFFE/$FILE/Mauritius%202003-04.pdf?openelement.

[CR32] BAT. (2005). Social Report 2004/2005, Accessed Mar 15, 2009 from http://www.bat.com/group/sites/uk__3mnfen.nsf/vwPagesWebLive/DO6RZGHL/$FILE/medMD6EMJJX.pdf?openelement.

[CR33] BAT. undated-a. *Cora research: Stakeholder mapping and classification*. Bates numbers: 325105696–325105729. Accessed May 12, 2008 from http://legacy.library.ucsf.edu/tid/abs62a99.

[CR61] BAT. undated-b. *Stakeholder mapping and classification*. Bates numbers: 760024456–760024463. Accessed May 12, 2008 from http://legacy.library.ucsf.edu/tid/wmj45a99.

[CR164] BAT. undated-c. *British American tobacco: Cora competency framework*. Bates numbers: 760078459–760078516. Accessed May 12, 2008 from http://legacy.library.ucsf.edu/tid/cal45a99.

[CR165] BAT. undated-d. *Business principles and framework for CSR*. Accessed Jun 20, 2009 from http://www.bat.com/oneweb/framework.nsf/F/MB1?opendocument.

[CR154] BAT. undated-e.* Second hand smoke*. Accessed Feb 15, 2011 from http://www.bat.com/group/sites/uk__3mnfen.nsf/vwPagesWebLive/DO52AMJ4?opendocument&SKN=1.

[CR36] BAT. undated-f. *How can you justify selling a product that is harmful to people’s health?* Accessed Feb 15, 2011 from http://www.bat.com/group/sites/uk__3mnfen.nsf/vwPagesWebLive/DO53DF8B?opendocument&SKN=1&TMP=1.

[CR155] BAT. undated-g. *Balanced regulation*. Accessed Feb 15, 2011 from http://www.bat.com/group/sites/uk__3mnfen.nsf/vwPagesWebLive/DO75TD4P?opendocument&SKN=1&TMP=1.

[CR156] BAT. undated-h. *Regulation and lobbying*. Accessed Feb 15, 2011 from http://www.bat.com/group/sites/uk__3mnfen.nsf/vwPagesWebLive/DO726J59?opendocument&SKN=1&TMP=1.

[CR35] BAT. undated-i. *British–American tobacco corporate brand and reputation management*. Bates numbers: 321483521–321483525. Accessed Oct 10, 2009 from http://legacy.library.ucsf.edu/tid/mle71a99.

[CR167] Baumberg, B. undated. *Convenient truths: An empirical investigation of moral perceptions of ‘alcohol industry’ employees, and the implications for theories of moral decision*-*making*, Draft Institute of Alcohol Studies Working Paper.

[CR166] Beelman, M. S., Campbell, D., Ronderos, M. T., & Schelzig, E. J. (2000). *Major tobacco multinational implicated in cigarette smuggling, tax evasion, documents show*. Investigative report. Washington D.C: International Consortium of Investigative Journalists, The Centre for Public Integrity.

[CR168] Business in the Community: undated, Directory of Members: Aerospace and Defence, Accessed Dec 20, 2010 from http://www.bitc.org.uk/applications/members_directory/sector.rm?name=Aerospace%20%26%20Defence.

[CR38] Bovens M, ‘t Hart P, Dekker S, Verheuvel G, Anheier H (1999). The politics of blame avoidance. When things go wrong.

[CR39] Broughton, M. (1998). Corporate social responsibility: A strategic imperative for British American Tobacco. Bates numbers: 321712287–321712288. Accessed Jun 13, 2008 from http://legacy.library.ucsf.edu/tid/qnf44a99.

[CR40] Burlinghame DF, Young DR (1996). Corporate philanthropy at the crossroads.

[CR41] Burton, B., & Rowell, A. (2002). British American tobacco’s socially responsible smoke screen, *PR Watch*, *9*(4): 6–12, http://www.prwatch.org/files/pdfs/prwatch/prwv9n4.pdf.

[CR42] Callard C (2010). Follow the money: How the billions of dollars that flow from smokers in poor nations to companies in rich nations greatly exceed funding for global tobacco control and what might be done about it. Tobacco Control.

[CR169] Campaign against the arms trade: Undated, other SFO investigations into BAE, Accessed Sep 25, 2009 from http://www.controlbae.org/background/sfo.php.

[CR43] Campaign for Tobacco Free Kids. (2001). *Illegal pathways to illegal profits: The big cigarette companies and international smuggling*, Accessed Mar 23, 2009 from http://www.tobaccofreekids.org/campaign/global/framework/docs/Smuggling.pdf.

[CR44] Campbell, D. (2004). Lost in transit, New Internationalist. 370, 21st July 2004.

[CR45] Campbell D, Slack R (2008). Corporate ‘philanthropy strategy’ and ‘strategic philanthropy some insights from voluntary disclosures in annual reports’. Business and Society.

[CR46] Canning, A. (1999). Notes from Alison Canning to Adrian Marshall regarding brand development strategy, Bates number: 321483520. Accessed Oct 10, 2009 from http://legacy.library.ucsf.edu/tid/lle71a99.

[CR47] Carter SM, Chapman S (2003). Smoking, disease and obdurate denial: The Australian tobacco industry in the 1980s. Tobacco Control.

[CR48] Centers for Disease Control and Prevention (2002). Annual smoking-attributable mortality, years of potential life lost, and economic costs: United States, 1995–1999. Morbidity and Mortality Weekly Report.

[CR49] Cohen S (1993). Human rights and crimes of the state: the culture of denial. Australian and New Zealand Journal of Criminology.

[CR50] Cohen S (2001). States of denial: Knowing about atrocities and suffering.

[CR51] Cohen JE, Milio N, Rozier RG, Ferrence R, Ashley MJ, Goldstein AO (2000). Political ideology and tobacco control. Tobacco Control.

[CR52] Cohen D, Prusak L (2001). In good company: How social capital makes organizations work.

[CR53] Collin J, Gilmore A (2002). Corporate (Anti)social (Ir)responsibility: Transnational tobacco companies and the attempted subversion of global health policy. Global Social Policy.

[CR54] Commission of the European Communities. (2001). *Promoting a European framework for Corporate Social Responsibility*, COM(2001) 366 final, Brussels.

[CR55] Commission of the European Communities. (2006). Communication from the Commission to the European Parliament, the Council and the European Economic and Social Committee Implementing the Partnership for Growth and Jobs: Making Europe a Pole of Excellence, COM(2006) 136 final, Brussels.

[CR56] Conklin JE (1977). Illegal but not criminal: Business crime in America, corporate in America.

[CR57] Corporate Watch (2006). What’s wrong with corporate social responsibility?.

[CR58] Cressey DR (1953). Other people’s money: A study of the social psychology of embezzlement.

[CR59] Crowther D, Njavro D, Krkac K (2006). Standards of corporate social responsibility: Convergence within the European Union. Business ethics and corporate social responsibility.

[CR60] Cummings KM, Morley CP, Hyland A (2002). Failed promises of the cigarette industry and its effect on consumer misperceptions about the health risks of smoking. Tobacco Control.

[CR62] Dearlove J, Bialous S, Glantz SA (2002). Tobacco industry manipulation of the hospitality industry to maintain smoking in public places. Tobacco Control.

[CR63] Diethelm P, McKee M (2009). Denialism: What is it and how should scientists respond?. European Journal of Public Health.

[CR64] Entman RM (1993). Framing: Toward clarification of a fractured paradigm. Journal of Communication.

[CR65] Fooks, G., & Gilmore, A. (2011). *From lobbying to corporate responsibility: the evolution of tobacco industry political activity*, European Conference on Tobacco or Health, 27th–30th March 2011, Netherlands: Amsterdam.

[CR68] Fooks, G. Collin, J., Smith, K., & Gilmore, A. (2009). *Revitalising the political authority of the tobacco industry; British American tobacco’s CSR programme*, 14th World Conference on Tobacco or Health, 11th–14th March, 2009, Mumbai, India.

[CR66] Fooks G, Gilmore A, Smith K, Collin J, Holden C, Lee K (2011). Corporate social responsibility and access to policy élites: An analysis of tobacco industry documents. PLoS Med.

[CR67] Forster N, Cassell C, Symon G (1994). The analysis of company documentation. Qualitative methods in organizational research: A practical guide.

[CR69] Gilmore AB, Collin J, McKee M (2006). British American tobacco’s erosion of health legislation in Uzbekistan. British Medical Journal.

[CR70] Gilmore A, Fooks G, McKee M (2009). The international monetary fund and tobacco: A product like any other?. International Journal of Health Services.

[CR71] Gilmore A, Fooks G, McKee M (2011). A review of the impacts of tobacco industry privatization: Implications for policy. Global Public Health.

[CR72] Glantz SA, Slade J, Bero LA, Hanauer P, Barnes DE (1996). The cigarette papers.

[CR73] Goffman E (1959). The presentation of self in everyday life.

[CR74] Gray GC (2006). The regulation of corporate violations: Punishment, compliance, and the blurring of responsibility. British Journal of Criminology.

[CR75] Grüning T, Gilmore AB, McKee M (2006). Tobacco industry influence on science and scientists in Germany. American Journal of Public Health.

[CR76] Haltom, W., McCann, M. & Fisher, S. (2009). Enduring effects of tobacco litigation, Paper presented at the annual meeting of The Law and Society Association, Grand Hyatt, Denver, Colorado, 25.05.09, Accessed Mar 24, 2011 from http://www.allacademic.com/one/www/www/index.php?cmd=Download+Document&key=unpublished_manuscript&file_index=2&pop_up=true&no_click_key=true&attachment_style=attachment&PHPSESSID=77af5a13870809ce98c2c971efc998de.

[CR77] Hardell L, Walker MJ, Walhjalt B, Friedman LS, Richter ED (2007). Secret ties to industry and conflicting interests in cancer research. American Journal of Industrial Medicine.

[CR78] Hedley, D. (2007). Consolidation endgame in sight: But is there one more big throw of the dice? Accessed Jun 18, 2009 from http://www.euromonitor.com/Consolidation_endgame_in_sight_but_is_there_one_more_big_throw_of_the_dice.

[CR79] Henriksen L, Dauphinee AL, Wang Y, Fortmann SP (2006). Industry sponsored anti-smoking ads and adolescent reactance: Test of a boomerang effect. Tobacco Control.

[CR80] Herter, U. (1995). BATCo Speech by Ulrich Herter. Bates numbers: 700845465–73. Accessed Apr 19, 2008 from http://legacy.library.ucsf.edu/tid/mhr14a99.

[CR81] Heslin PA, Ochoa JD (2008). Understanding and developing strategic corporate social responsibility. Organizational Dynamics.

[CR82] Hill MR (1993). Archival strategies and techniques, qualitative research methods.

[CR83] Hoggan J, Littlemore R (2009). Climate cover-up: The crusade to deny global warming.

[CR84] Holden C, Lee K (2009). Corporate power and social policy: The political economy of the transnational tobacco companies. Global Social Policy.

[CR85] Hopkins DP, Razi S, Leeks KD, Kalra GP, Chattopadhyay SK, Soler RE, Task Force on Community Preventive Services (2010). Smokefree policies to reduce tobacco use: A systematic review. American Journal of Preventive Medicine.

[CR86] Hurt RD, Ebbert JO, Muggli ME, Lockhart NJ, Robertson CR (2009). Open doorway to truth: Legacy of the Minnesota tobacco trial. Mayo Clinic Proceedings.

[CR87] Jacobson PD, Wasserman J, Anderson JR (1997). Historical overview of tobacco legislation and regulation. Journal of Social Issues.

[CR88] Jacoby WG (2000). Issue framing and public opinion on government spending. American Journal of Political Science.

[CR89] Janofsky, M. (1994). Tobacco industry tries a new pitch: Openness. New york: The New York Times.

[CR90] Kaye M (1995). Organisational myths and storytelling as communication management. Journal of Management and Organization.

[CR91] Klockars C (1974). The professional fence.

[CR92] Konovsky MA, Jaster F (1989). Blaming the victim’ and other ways business men and women account for questionable behavior. Journal of Business Ethics.

[CR93] Lader, D. (2007). *Smoking*-*related Behaviour and Attitudes, 2006*, A report on research using the National Statistics Omnibus Survey produced on behalf of the Information Centre for health and social care, Omnibus Survey Report No. 32, Accessed Feb 17, 2011 from http://www.statistics.gov.uk/downloads/theme_health/smoking2006.pdf.

[CR94] Landman A, Ling PM, Glantz SA (2002). Tobacco industry youth smoking prevention programs: Protecting the industry and hurting tobacco control. American Journal of Public Health.

[CR95] Larrinaga-Gonzalez C, Bebbington J (2001). Accounting change or institutional appropriation?: A case study of the implementation of environmental accounting. Critical Perspectives on Accounting.

[CR96] Leavell NR, Muggli ME, Hurt RD, Repace J (2006). Blowing smoke: British American tobacco’s air filtration scheme. British Medical Journal.

[CR97] Leigh, D. & Evans, R. (2009), Fraud Office seeks BAE’s prosecution over bribery, *The Guardian*, 1.10.09.

[CR98] Mamudu HM, Hammond R, Glantz SA (2008). Project Cerberus: Tobacco industry strategy to create an alternative to the framework convention on tobacco control. American Journal of Public Health.

[CR99] Marshall, A. (2000a).* First draft of Europe speech*. Bates numbers: 325418914–325418926. Accessed May 10, 2008 from http://bat.library.ucsf.edu/tid/vfs62a99.

[CR100] Marshall, A. (2000b). Untitled. 1.03.00. Bates numbers: 325050319–325050340. Accessed May 10, 2008 from http://legacy.library.ucsf.edu/tid/pap14a99.

[CR101] Marshall, T. R. (2010). *Lost public opinion: American public opinion and the 1964 surgeon general’s report on smoking*, Paper presented at the Western Political Science Association annual convention, San Francisco, California, April 2010, Accessed Mar 24, 2011 from http://papers.ssrn.com/sol3/Delivery.cfm/SSRN_ID1582260_code238096.pdf?abstractid=1580968&mirid=3.

[CR102] Mathers CD, Loncar D (2006). Projections of global mortality and burden of disease from 2002 to 2030. PLoS Medicine.

[CR103] Maxwell JW, Lyon TP, Hackett SC (2000). Self-regulation and social welfare: The political economy of corporate environmentalism. Journal of Law and Economy.

[CR104] McCombs ME, Shaw DL, Weaver DH (1997). Communication and democracy: Exploring the intellectual Frontiers in agenda-setting theory.

[CR105] McCulloch J, Tweedale G (2008). Defending the indefensible: The global asbestos industry and its fight for survival.

[CR106] McDaniel PA, Malone RE (2009). The role of corporate credibility in legitimizing disease promotion. American Journal of Public Health.

[CR107] McGarity TO, Wagner WE (2008). Bending science: How special interests corrupt public health research.

[CR108] McGraw KM (1990). Avoiding blame: An experimental investigation of political excuses and justifications. British Journal of Political Science.

[CR109] Menashe CL, Siegel M (1998). The power of frame: An analysis of newspaper coverage of tobacco issues: United States, 1985–1996. Journal of Health Communication.

[CR110] Millson, S. (1999).* WHO tobacco free initiative*. Bates numbers: 321858071–321858074. Accessed May 27, 2008 from http://legacy.library.ucsf.edu/tid/tiu50a99.

[CR111] Minor WW (1981). Techniques of neutralization: A reconceptualization and empirical examination. Journal of Research in Crime and Delinquency.

[CR112] Muggli ME, Kelley Lee K, Quan Gan Q, Ebbert JO, Hurt RD (2008). Efforts to reprioritise the Agenda in China: British American tobacco’s efforts to influence public policy on secondhand smoke in China. PLoS Med.

[CR113] Nathanson C (2005). Collective actors and corporate targets in tobacco control: A cross-national comparison. Health Education and Behavior.

[CR114] National Statistics. (2000) *Living in Britain*, Accessed Feb 17, 2011 from http://www.statistics.gov.uk/lib2000/viewerChart523.html.

[CR115] O’Dwyer B (2003). Conceptions of corporate social responsibility: The nature of managerial capture. Accounting, Auditing and Accountability Journal.

[CR116] Office for National Statistics. (2009). *Smoking-related behaviour and attitudes, 2008/09*, Opinions Survey Report No. 40, Accessed Apr 19, 2010http://www.statistics.gov.uk/downloads/theme_health/smoking2008-9.pdf.

[CR117] Office for National Statistics. (2011). *Two-thirds of smokers say they want to quit the habit*, Press Release http://www.statistics.gov.uk/pdfdir/nsd0311.pdf, 15.03.11.

[CR118] Oliver, J. (1998).* Corporate responsibilities programme*. Bates numbers: 322121448–322121455. Accessed May 05, 2008 from http://legacy.library.ucsf.edu/tid/yoz82a99.

[CR119] Ong EK, Glantz SA (2001). Constructing “sound science” and “good epidemiology”: Tobacco, lawyers, and public relations firms. American Journal of Public Health.

[CR120] Opukah, S. (1997).* Email from Shabanji Opukah to AMESCA Area Directors, General Managers and CORA Managers outlining CORA plans 1997–99*. Bates numbers: 800098725–800098728. Accessed May 4, 2008 from http://legacy.library.ucsf.edu/tid/hvj44a99.

[CR121] Orlitzky M, Schmidt FL, Rynes SL (2003). Corporate social and financial performance: A meta-analysis. Organization Studies’.

[CR122] Owen DL, Gray R, Bebbington J (1997). Green accounting: Cosmetic irrelevance or radical agenda for change?. Asia-Pacific Journal of Accounting.

[CR123] Owen DL, Swift TA, Humphrey C, Bowerman M (2000). The new social audits: Accountability, managerial capture or the agenda of social champions?. European Accounting Review.

[CR124] Palazzo G, Richter U (2005). CSR business as usual? The case of the tobacco industry. Journal of Business Ethics.

[CR125] Pearce F, Tombs S (1998). Toxic capitalism: Corporate crime and the chemical industry.

[CR126] Piquero N, Tibbetts S, Blankenship M (2005). Examining the role of differential association and techniques of neutralization in explaining corporate crime. Deviant Behavior.

[CR127] Porter ME, Kramer MR (2002). The competitive advantage of corporate philanthropy. Harvard Business Review.

[CR128] Prideaux, M. (2000). *Meeting reasonable public expectations of a responsible tobacco company*. Bates numbers: 325049576–595. Accessed May 11, 2008 from http://legacy.library.ucsf.edu/tid/hwg61a99.

[CR129] Prideaux, M. & Smith, J. (1999). CORA planning guidelines for 2000. 02.07.99. Bates numbers: 321699794–321699799. Accessed sep 10, 2009 from http://legacy.library.ucsf.edu/tid/xyj34a99.

[CR130] Proctor, C. J. (1995a). *Project battalion: The role of corporate affairs*. 27.05.95. Bates numbers: 503106864–503106873. Accessed oct 26, 2009 from http://legacy.library.ucsf.edu/tid/fda04a99.

[CR131] Proctor, C. J. (1995b). *Fax from Chris Proctor (of the Smoking Issues Department) to Stuart Chalfen*. Bates numbers: 500820183–92. http://legacy.library.ucsf.edu/tid/xll04a99, 19.04.08.

[CR170] *R and BAE systems PLC*, Southwark Crown Court, 1 English Grounds, Southwark, London, 21/12/10, Accessed Jan 28, 2001 from http://www.judiciary.gov.uk/Resources/JCO/Documents/Judgments/r-v-bae-sentencing-remarks.pdf.

[CR132] Research International. (1997a). *British-American tobacco opinion benchmark study level 2: UK staff findings*. Bates numbers: 700809061–700809126. Accessed May 13, 2008 from http://legacy.library.ucsf.edu/tid/qgr14a99.

[CR133] Research International. (1997b). *British-America tobacco opinion benchmark study level 1 market findings*. Bates numbers: 321268107–321268354. Accessed May 13, 2008 from http://legacy.library.ucsf.edu/tid/ncz13a99.

[CR134] Ritch WA, Begay ME (2001). Strange bedfellows: The history of collaboration between the Massachusetts restaurant association and the tobacco industry. American Journal of Public Health.

[CR135] Rosner D, Markowitz G (1994). Deadly dust: Silicosis and the politics of occupational disease in twentieth-century America.

[CR136] Rosner D, Markowitz G (2006). Deadly dust: Silicosis and the ongoing struggle to protect worker’s health.

[CR137] Saiia DH (2001). Philanthropy and corporate citizenship: Strategic philanthropy is good corporate citizenship. Journal of Corporate Citizenship.

[CR138] Saiia DH, Carroll AB, Buchholtz AK (2003). Philanthropy as strategy: When corporate charity begins at home. Business and Society.

[CR139] Schneider SK, Jacoby WG (2005). Elite discourse and American public opinion: The case of welfare spending. Political Research Quarterly.

[CR140] Sebrié EM, Glantz SA (2007). Attempts to undermine tobacco control: Tobacco industry “youth smoking prevention” programs to undermine meaningful tobacco control in Latin America. American Journal of Public Health.

[CR142] Smith KE, Fooks G, Collin J, Weishaar H, Mandal S, Gilmore AB (2010). Working the system: British American tobacco’s influence on the European Union Treaty and its implications for policy: An analysis of internal tobacco industry documents. PLoS Medicine.

[CR143] Smith KE, Gilmore AB, Fooks G, Collin J, Weishaar H (2009). Tobacco industry attempts to undermine Article 5.3 and the “good governance” trap. Tobacco Control.

[CR171] SourceWatch. undated. *Tobacco industry public relations strategies*, Accessed Nov 4, 2010 from http://www.sourcewatch.org/index.php?title=Tobacco_industry_public_relations_strategies.

[CR144] Stauber J, Rampton S (1995). Toxic sludge is good for you.

[CR145] Sykes GM, Matza D (1957). Techniques of neutralization: A theory of delinquency. American Sociological Review.

[CR146] Szwajkowski E (1992). Accounting for organizational misconduct. Journal of Business Ethics.

[CR147] Tesler L, Malone RE (2008). Corporate philanthropy, lobbying and public health policy. American Journal of Public Health.

[CR148] Test Research. (2000).* British American tobacco stakeholders analysis. A research study conducted for BAT by Test Research*. Bates numbers: 325105859–325105876. Accessed May 11, 2008 from http://legacy.library.ucsf.edu/tid/axc14a99.

[CR149] Thompson DF (1980). Moral responsibility of public officials: The problem of many hands. American Political Science Review.

[CR150] Thomson G (2005). Trust us we’re socially responsible. The truth behind British American Tobacco NZ’s Corporate Social Responsibility reports.

[CR151] Thorne D, Ferrell OC, Ferrell L (2003). Business and society: A strategic approach to corporate citizenship.

[CR4] Tobacco Free Initiative. (2006). Facts and figures about tobacco. Accessed Sep 20, 2009 form www.who.int/entity/tobacco/fctc/tobacco%20factsheet%20for%20COP4.pdf.

[CR152] Tucker E (2006). Working disasters: Politics of recognition and response.

[CR153] Tweedale G (2000). Magic mineral to killer dust.

[CR1] U.S. Newswire. (1997). *Poll shows anger at tobacco industry skyrocketing*, 27th March 1997.

[CR157] Vaughan E, Seifert M (1992). Variability in the framing of risk issues. Journal of Social Issues.

[CR158] Wagner WE, Steinzor RI (2006). Rescuing science from politics: Regulation and the distortion of scientific research.

[CR159] Wallack L, Dorfman L, Jernigan D, Themba M (1993). Media advocacy and public health: Power for prevention.

[CR160] Warner KE, Fulton GA (1995). Importance of tobacco to a country’s economy: An appraisal of the tobacco industry’s economic argument. Tobacco Control.

[CR161] Weishaar, H., Grüning, T., Smith, E. A., Mandal, S., Gilmore, A. & Collin, J. (under review) Commercial sector opposition to global health governance: Transnational tobacco corporation strategies to influence the WHO’s Framework Convention on Tobacco Control, *PLoS Medicine*. 10.1371/journal.pmed.1001249 PMC3383743 22745607

[CR162] World Bank (1999). Curbing the epidemic: Governments and the economics of tobacco control.

[CR163] World Health Organization. (2004). *Tobacco Industry and Corporate Social Responsibility… an Inherent Contradiction*, Accessed Jun 3, 2009 from http://repositories.cdlib.org/tc/whotcp/CSR.

[CR172] Yang J, Malone RE (2008). Working to shape what society’s expectations of us should be: Philip Morris’s societal alignment strategy. Tobacco Control.

